# Core of sensory gating deficits in first-episode schizophrenia: attention dysfunction

**DOI:** 10.3389/fpsyt.2023.1160715

**Published:** 2023-04-26

**Authors:** Yushen Ding, Qing Tian, Wenpeng Hou, Zhenzhu Chen, Zhen Mao, Qijing Bo, Fang Dong, Chuanyue Wang

**Affiliations:** ^1^Beijing Key Laboratory of Mental Disorders, Beijing Institute for Brain Disorders Center of Schizophrenia, Beijing Anding Hospital, Capital Medical University, Beijing, China; ^2^Advanced Innovation Center for Human Brain Protection, Capital Medical University, Beijing, China; ^3^Suzhou Guangji Hospital, The Affiliated Guangji Hospital of Soochow University, The Institute of Mental Health, Suzhou, China

**Keywords:** schizophrenia, attention, cognitive function, MCCB, modified prepulse inhibition

## Abstract

**Background:**

Sensory gating deficits are a common feature of schizophrenia and may be indicative of higher-order psychopathological impairments. It has been proposed that incorporating subjective attention components into prepulse inhibition (PPI) measures may improve the accuracy of assessing these deficits. This study aimed to investigate the relationship between modified PPI and cognitive function, with a specific focus on subjective attention, to gain a better understanding of the underlying mechanisms of sensory processing deficits in schizophrenia.

**Methods:**

Fifty-four unmedicated first-episode schizophrenia (UMFE) patients and 53 healthy controls participated in this study. The modified Prepulse Inhibition paradigm, including Perceived Spatial Separation PPI (PSSPPI) and Perceived Spatial Colocation PPI (PSCPPI), was used to evaluate sensorimotor gating deficits. Cognitive function was assessed in all participants using the Chinese version of the MATRICS Consensus Cognitive Suite Test (MCCB).

**Results:**

UMFE patients had lower MCCB scores and deficient PSSPPI scores than healthy controls. PSSPPI was negatively correlated with total PANSS scores and positively correlated with the speed of processing, attention/ vigilance, and social cognition. Multiple linear regression analysis showed that the PSSPPI at 60 ms had a significant effect on attentional/ vigilance and social cognition, even after controlling for gender, age, years of education, and smoking.

**Conclusion:**

The study revealed notable impairments in sensory gating and cognitive function in UMFE patients, best reflected by the PSSPPI measure. Specifically, PSSPPI at 60 ms was significantly associated with both clinical symptoms and cognitive performance, suggesting that PSSPPI at 60 ms may capture psychopathological symptoms related to psychosis.

## 1. Introduction

Schizophrenia is a severe mental disease characterized by positive symptoms, negative symptoms, and cognitive impairment (1), with a global prevalence of 1% (2). Individuals with schizophrenia commonly display abnormal information processing, whereby they have difficulty inhibiting irrelevant stimuli. This can result in an overload of irrelevant information in their consciousness, leading to thought disorders and other core symptoms associated with schizophrenia (3). Some authors have proposed that changes in PPI (prepulse inhibition) in patients with schizophrenia may be a cause of the characteristic sensory information overload of this disease. This hypothesis is based on the idea that PPI can reflect the ability to regulate the amount of sensory information processed continuously and plays a crucial role in filtering relevant and irrelevant sensory information (3, 4). Over the past few decades, numerous studies have demonstrated the presence of defects in PPI among patients with schizophrenia (5, 6), which has established PPI as a potential phenotype of the disorder (7, 8).

PPI is a classical gating index that involves presenting a low-intensity pre-stimulus (known as the “prepulse”) 10–500 ms before the startle stimulus (“pulse”), resulting in a reduction of the startle response (9, 10). PPI is an automatic processing process, and the corresponding neural circuits are primarily located in the brainstem, including the auditory mesencephalic inferior colliculus, the deep layer of the superior colliculus, and the tegmental nucleus of the pontine foot. These circuits have extensive neural connections with the sensory cortex, joint cortex, motor system, and limbic system, providing a biological basis for the top-down regulation of developed cognitive processes such as PPI attention (11). The modified PPI paradigm utilizes the condition of perceptual space separation to isolate the subjective sensory sound direction when the background noise is distinct from the perceived direction of the prepulse stimulus, has been successfully constructed for inducing spatial selective attention(i.e., the perceived spatial separation PPI paradigm) (12). There is substantial evidence indicating that introducing attention to the prepulse significantly increases PPI (13–15).

The relationship between PPI and cognitive function remains inconclusive in existing research. Mixed findings have been reported regarding the relationship between PPI and continuous performance task (CPT). While two studies found no correlation between PPI and behavioral measures of CPT in healthy controls and patients with schizophrenia (16, 17), another study found that higher PPI was associated with better CPT performance in healthy controls (18). Kirsty et al. (19) used novel methods for the measurement of PPI, introducing instructed PPI tasks, and found a correlation between PPI and attention and memory tasks under different conditions. The authors also showed that these relationships seem to be mediated by common attentional processes active within both PPI and cognitive tasks. Previous studies have found that this circuit overlaps with related areas of psychopathological manifestations in patients with schizophrenia, such as thought disorders (20), social cognition (21), and emotional perception deficits (22). Therefore, modified PPI can indirectly indicate high-order psychopathological impairments.

Attentional impairment may be at the core of all cognitive function impairments, with meta-analyses shuowing a medium to large effect size (23–25). Recent studies have also found attentional deficits in first-episode schizophrenia patients, and although these deficits improve over time, but still do not reach the level of healthy controls (26, 27). There is also evidence suggesting that schizophrenia patients have deficits in selective attention (28, 29). Considering these factors, we hypothesized that the modified PPI paradigm would be a more robust measure of impairment and could potentially provide a more sensitive measure of specific cognitive variables that are important in first-episode schizophrenia.

The cognitive evaluation tools used in assessing cognitive function in schizophrenia are contradictory in existing studies, and the domains of cognitive function assessment are inadequate and influenced by factors such as drug treatment and the course of the disease. For instance, patients with schizophrenia who received medication, particularly those who received second-generation antipsychotic drugs, had higher PPI compared to patients who did not receive medication (30). Although there is coherent research outcomes on the PPI deficits, there are few analyses on modified PPI, particularly on the association between it and cognitive function (31). To address this gap, this study utilized a standardized cognitive evaluation tool to assess the cognitive function of unmedicated first-episode schizophrenia patients and evaluate the relationship between prepulse inhibition and cognitive function. The aim of this study is to provide a better understanding of the correlation between PPI and cognition.

## 2. Materials and methods

### 2.1. Participants

The unmedicated first-episode schizophrenia group (UMFE) was recruited from both the outpatient and inpatient departments of Beijing Anding Hospital. Inclusion criteria were as follows: age between 15 and 45 years; normal hearing without any prior auditory system diseases; at least 9 years of education, and a Simple Wechsler intelligence test score of 80 or greater. The Chinese version of the MINI International Neuropsychiatric Interview (MINI) (6.0) was used to diagnose patients with schizophrenia according to the diagnostic criteria outlined in the Fifth edition of the Diagnostic and Statistical Manual of Mental Disorders (DSM-5). Additionally, patients with a duration of illness less than 5 years, who had not received medication or continuous application of antipsychotics since the onset of symptoms for fewer than 2 weeks, were included.

Exclusion criteria were as follows: serious abnormalities found in physical evaluation, laboratory biochemical indicators, or electroencephalogram (EEG), electrocardiogram (ECG); pregnant or lactating women; patients in a state of extreme excitement and impulsivity as determined by their treating physician; patients who were deemed likely to commit suicide or violence during the study; patients who had received electroconvulsive or magnetic stimulation treatment within 6 months prior to recruitment.

The healthy control group was matched for age, gender, and years of education with the patient group and had no personal or family history of mental diseases.

### 2.2. Sample size estimate

Sample size calculations were performed using online software and by consulting relevant literature and prior similar studies. Based on previous research demonstrating a medium effect size (Cohen’s d = 0.6) in distinguishing between the two groups, a desired power of 1-β = 0.85, and a significance level of α = 0.05, a minimum sample size of 50 participants per group was determined.

### 2.3. Clinical and cognition assessments

A self-signed evaluation was used to collect general and disease-related information from the subjects. The Positive and Negative Syndrome Scale (PANSS) is a widely used clinical rating scale that measure the severity of symptoms in patients with schizophrenia (32). It consists of 30 items, 7 of which measure positive symptoms (e.g., hallucinations, delusions), 7 of which measure negative symptoms (e.g., blunted affect, social withdrawal), and 16 of which measure general psychopathology (e.g., anxiety, depression). Each item is rated on a scale of 1 (absent) to 7 (extreme), and the total score ranges from 30 to 210, with higher scores indicating more severe symptoms. We utilized the MATRICS Consensus Cognitive Battery (MCCB) tool to determine individual neuropsychological states (33). The Chinese version of the MCCB consists of 7 cognitive domains, and 9 tests were chosen to represent seven cognitive domains in the MCCB. Information processing speed, including Trail Making Test, Part A, symbol coding subtest, and animal naming; Attention/vigilance, including the Continuous Performance Test; Working memory, including the spatial span subtest; Verbal learning, including the Hopkins Verbal Learning Test; Visual learning, including the Brief Visuospatial Memory Test; Reasoning and problem-solving, including the mazes subtest; Social cognition, including the Mayer-Salovey-Caruso Emotional Intelligence Test. The PANSS assessment was administered by experienced psychiatrists who underwent consistent training to ensure the accuracy and reliability of the results. In addition, trained psychologists administered the MCCB evaluation.

### 2.4. PPI measures

The modified PPI test was conducted in a shielded room using the Xeye Human Startle Reflex system. Two Ag/AgCl electrodes were attached to the pupil and lateral canthus of the right eye to record the electrical activity of the orbicularis oculi muscle, and the electrodes were grounded using the right mastoid. The PPI paradigm was tested under the conditions of perceptual spatial separation and colocation.

The stimulus parameters were as follows: the startle stimulus was a 40 ms 100 dB SPL(Sound Pressure Level, SPL) white noise, the prepulse stimulus was an 800 Hz narrowband noise, 150 ms 64 dB SPL, and the background noise was 60 dB SPL white noise. The interval of stimulation (ISI) between the prepulse and startle stimulus was 120 ms/ 60 ms, and the interaural delay of background noise between the left and right ear was 3 ms.

The stimulus sequence involved playing the startle stimulus 5 times. If the subjects captured the startle reflex normally, the experiment continued. Two 800 Hz narrowband noises were played with an ISI of 120 ms/60 ms. If the subject heard both sounds, the test continued later. The background noise and the prepulse stimulus were played, and the subjects were asked to identify the sound from the left or the right. After a few practice sessions, the correct rate was set to >90%, and the experiment proceeded with two blocks.

**Figure 1:**
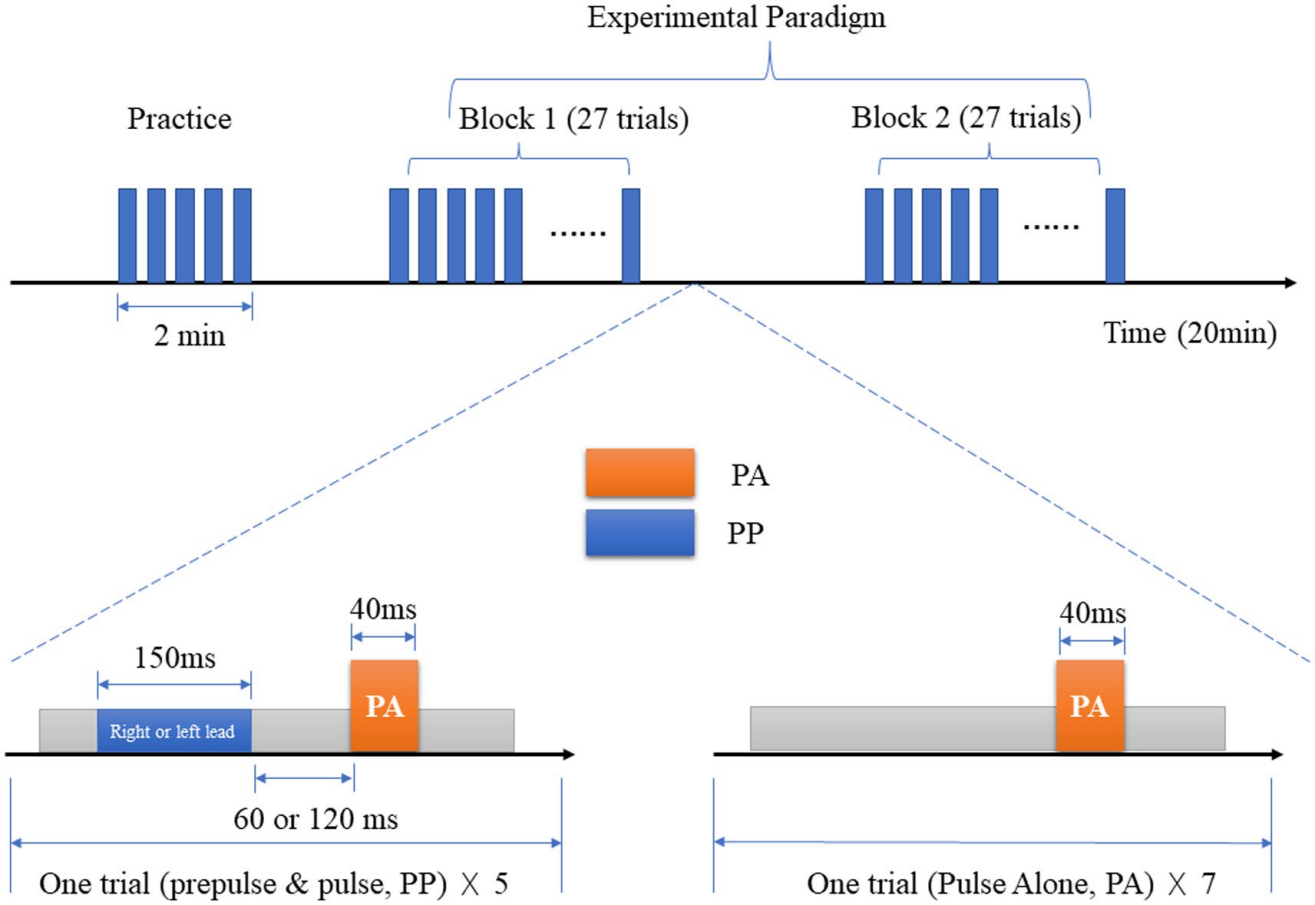
Modified experimental paradigm of prepulse inhibition.

In the first block, the background noise always came from the left with a total of 27 trials. In the second block, the background noise always came from the right with a total of 27 trials. Depending on the subjective feeling formed between the background noise and the prepulse stimulus, it was classified into perceptual space separation/colocation (PSSPPI/PSCPPI), resulting in a total of 4 types of prepulse stimulation (see Figure 1).

The PPI test was administered by experienced researchers who received consistent training to ensure accuracy and reliability.

### 2.5. Data processing

Each trial was evaluated to remove the electromyography (EMG) response caused by automatic blinking. The mean and maximum peak values of each trial’s sampling period were identified. A trial was considered valid if the maximum peak was greater than or equal to the sampling period mean multiplied by 4 and the sampling period mean was greater than or equal to the response period mean; otherwise, the trial was considered invalid., the trial was considered invalid. The maximum peak latency range was 350–850 ms. PPI was calculated using the formula: PPI = (1-pp/p) ^*^100%, where ‘p’ indicates the amplitude induced only under the condition of startle stimulation, and ‘pp’ represents the amplitude induced by prepulse stimulation plus startle stimulation.

### 2.6. Statistical analysis

Statistical analyses were performed using R4.1.1 software (Comprehensive R Archive Network, http://cran.rproject.org/). Continuous variables are presented as the mean (standard deviation), while categorical variables are presented as the frequency and percentage. Differences in demographic data, cognitive function, and modified PPI index between groups were assessed using the Chi-square test or independent sample t test. The relationship between the modified PPI index and PANSS score, as well as cognitive function, was assessed using Spearman correlation analysis, followed by multiple linear regression to further evaluate the correlation. Statistical significance was considered at the α = 0.05 level, and multiple comparisons were adjusted using the Bonferroni correction.

## 3. Results

### 3.1. Demographics and clinical characteristics

A total of 107 participants were included in the study, consisting of 54 unmedicated first-episode schizophrenia patients and 53 healthy controls. The two groups were matched in terms of age, gender, and years of education, and there was no significant difference in the number of smokers between them. Table 1 shows the PANSS score of the UMFE group.

**Table 1:** Social-demographical and clinical characteristics of all the study subjects.

	UMFE (*n* = 54)	HC (*n* = 53)	t/ χ2	*p* value
Age (year, mean (SD))	25.65 (8.41)	24.68 (6.37)	0.78	0.44
Gender, male (%)	23 (42.59%)	31 (58.49%)	2.70	0.10
Education level (year, mean (SD))	13.52 (3.70)	14.11 (3.21)	0.89	0.38
IQ	103.05(11.43)	113.12(11.99)	−4.40	<0.001
Family history, *n* (%)	12 (22%)	0 (0%)	13.27	<0.001
Employment rate, *n* (%)				
Unemployed	11 (20.37%)	0 (0%)	14.02	<0.001
Employed	24 (44.44%)	37 (69.81%)		
Student	19 (35.19%)	24 (30.19%)		
Smoking, *n* (%)	4 (7.41%)	9 (16.98%)	2.30	0.13
Age at onset (year, mean (SD))	23.96(8.42)	NA		
DUP (month, mean (SD))	10.94(16.17)	NA		
PANSS (mean (SD))				
Total score	76.52 (14.45)	NA		
Positive symptoms	21.33 (5.55)	NA		
Negative symptoms	16.98 (7.09)	NA		
General symptoms	38.20 (7.53)	NA		

UMFE, unmedicated first-episode schizophrenia. HC, healthy controls. IQ, Intelligence Quotient. DUP, duration of untreated psychosis. PANSS, the Positive and negative symptoms scale.

### 3.2. Cognitive function

After correcting for gender and age differences in MCCB cognitive assessments, the study found that the total cognitive scores and neurocognitive scores of the UMFE group were significantly lower than those of the healthy control group (all *p* < 0.01). However, there was no significant difference in social cognitive outcomes between the two groups (*p* = 0.18), as shown in Table 2.

**Table 2:** Cognitive function and PPI index results of participants.

	UMFE (*n* = 54)	HC (*n* = 53)	*t*	*p* value	*Cohen’s d* (95%CI)
MCCB					
Speed of processing	38.30 ± 10.89	45.81 ± 8.00	−4.07	<0.01	0.78(0.392–1.178)
Attention/vigilance	37.21 ± 12.48	46.09 ± 8.47	−4.31	<0.01	0.83(0.436–1.226)
Working memory	39.41 ± 10.26	47.53 ± 7.18	−4.75	<0.01	0.92(0.517–1.314)
Verbal learning	42.75 ± 10.43	48.68 ± 8.77	−3.19	<0.01	0.62(0.227–1.003)
Visual learning	42.92 ± 13.56	48.30 ± 9.19	−2.40	0.02	0.45(0.069–0.836)
Reasoning and problem-solving	39.95 ± 12.26	44.66 ± 11.15	−2.08	0.04	0.40(0.019–0.785)
Social cognition	38.61 ± 12.47	41.51 ± 9.84	−1.34	0.18	0.26(−0.123–0.638)
Overall composite	39.22 ± 9.00	46.37 ± 6.93	−4.61	<0.01	0.89(0.492–1.286)
PPI					
PA	58.56 ± 36.33	71.11 ± 31.98	−1.90	0.06	0.37(−0.016–0.749)
Latency	451.5 ± 71.21	465.51 ± 71.19	−1.03	0.31	0.20(−0.183–0.577)
PSCPPI 60	27.78 ± 21.18	33.37 ± 15.92	−1.55	0.13	0.30(−0.083–0.679)
PSSPPI 60	27.94 ± 21.99	42.35 ± 17.34	−3.77	<0.01	0.73(0.336–1.118)
PSCPPI 120	24.17 ± 23.81	32.07 ± 11.77	−1.95	0.05	0.42(0.036–0.803)
PSSPPI 120	25.72 ± 23.73	39.67 ± 15.42	−3.61	<0.01	0.70(0.305–1.086)

PSCPPI, Perceived spatial colocation PPI. PSSPPI, Perceived spatial separation PPI. UMFE, unmedicated first-episode schizophrenia. HC, healthy control.

### 3.3. Prepulse inhibition

**Figure 2:**
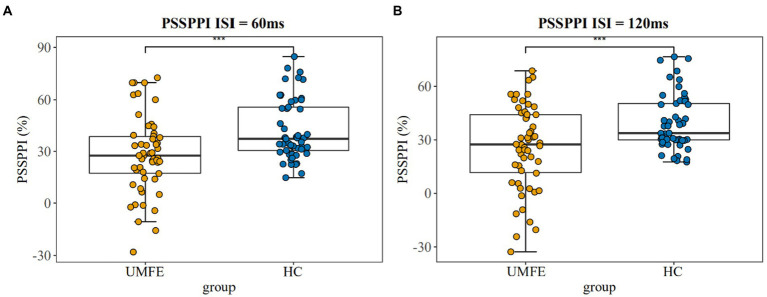
Prepulse Inhibition of perceptual space separation at 60 ms intervals (Left); Prepulse Inhibition of perceptual space separation at 120 ms intervals (Right). UMFE: unmedicated first-episode schizophrenia, HC: healthy control. ^***^p value <0.001.

There were no significant differences between the two groups in terms of the amplitude of startle stimulation and the latency of maximum amplitude. The UMFE group had lower scores than the healthy control group in the PPI index of perceptual spatial separation (PSSPPI) at both time intervals, while there was no significant difference in perceptual space colocation (PSCPPI) between the two groups at both the 60 ms and 120 ms intervals. The effect size of PSSPPI were 0.73 (Cohen’s d = 0.73, 95% CI [0.34–1.12]) at 60 ms and 0.70 (Cohen’s d = 0.70, 95% CI [0.31–1.09]) at 120 ms, with a statistically significant difference between the two groups (*p* < 0.01 for both). These results suggest that the UMFE group had impaired sensorimotor gating compared to the healthy control group. Table 2 and Figure 2 provide more details on these findings.

### 3.4. Correlation between Prepulse inhibition and clinical symptoms, cognitive function

Spearman correlation analysis showed that in the UMFE group, the PSSPPI at the 60 ms interval had significant correlations with some clinical symptoms and cognitive function. Specifically, it was negatively correlated with the PANSS total score (*r* = −0.27, *p* = 0.049), and positively correlated with speed of processing (*r* = 0.27, *p* = 0.044), attention/vigilance (*r* = 0.27, p = 0.049), and social cognition (*r* = 0.27, *p* = 0.048). However, no significant correlations were found between PSSPPI at the 120 ms interval and clinical symptoms or cognitive function. In the healthy control group, there were no significant correlations were found between PSSPPI and cognitive function. Table 3 shows correlation coefficients (*r*) between PSSPPI and clinical symptoms and cognitive function in the UMFE group, as well as between PSSPPI and cognitive function in the healthy control group. After Bonferroni correction, none of these results remained significant.

**Table 3:** Correlations between PSSPPI and clinical symptoms/cognitive function in UMFE group, and with cognitive function in HC groups.

	UMFE (n = 54)		HC (n = 53)	
	PSSPPI 60	PSSPPI 120	PSSPPI 60	PSSPPI 120
PANSS				
Total score	−0.27^*^	−0.15	NA	NA
Positive symptoms	−0.1	−0.06	NA	NA
Negative symptoms	−0.22	−0.14	NA	NA
General symptoms	−0.24	−0.15	NA	NA
MCCB				
Speed of processing	0.27^*^	0.20	0.07	0.04
Attention/ vigilance	0.27^*^	0.08	0.06	0.08
Working memory	0.14	0.17	−0.17	0.01
Verbal learning	0.16	−0.01	0.07	0.02
Visual learning	0.10	0.08	−0.08	−0.12
Reasoning and problem-solving	0.23	0.12	−0.17	0.06
Social cognition	0.27^*^	0.06	−0.04	0.07
Overall composite	0.23	0.13	−0.02	0.10

UMFE, unmedicated first-episode schizophrenia. HC, healthy controls. PANSS, the Positive and negative symptoms scale. PSCPPI: Perceived spatial colocation PPI. PSSPPI: Perceived spatial separation PPI. *p*-value for spearman rank correlation analysis. NA: not application, *p<0.05

Furthermore, to verify the association between cognitive function and PSSPPI in UMFE group, a regression analysis was performed with the PSSPPI as the dependent variable and cognitive function as the independent variable. The potential confounding factors, such as gender, age, years of education, and smoking, were included in the regression model. Additionally, the variance inflation factor (VIF) method was employed to detect collinearity issues. If the VIF is less than 10, it is considered that there is no multicollinearity issue among all independent variables. Multiple linear regression analysis revealed that PSSPPI at 60 ms intervals had a significant impact on attention/ vigilance (*β* = 0.23, *p* = 0.02) and social cognition (*β* = 0.23, *p* = 0.04), but had no effect on other cognitive domains. Additionally, PSSPPI at 120 ms intervals did not significantly affect the overall cognitive score or any of the specific cognitive domains.

## 4. Discussion

### 4.1. Cognitive deficits in UMFE

This study provides further evidence of prevalent neurocognitive impairment among unmedicated patients with first-episode schizophrenia, which is consistent with previous research (34, 35). Specifically, our study found a large effect size for impaired working memory and attentional vigilance in UMFE patients compared to healthy controls. Interestingly, no deficits in social cognition were observed in this group, which contradicts the findings of most earlier studies (34, 36, 37). For example, Pablo et al. (38) reported that both first-episode and chronic schizophrenia were associated with social cognition deficits. A recent meta-analysis of cross-sectional studies on cognitive function in schizophrenia also found a large effect size for social cognition (SMD = 0.88) (25). In contrast, a large sample study of schizophrenia in China reported a smaller effect size (Cohen’s d = 0.42) for the social cognition domain (39). Additionally, in another study, patients with first-episode schizophrenia scored slightly lower on the MSCEIT test than the controls (SMD = −0.38) (40). Social cognition is widely recognized as a mediator between neurocognitive deficits and functional outcomes (41). However, this study did not find significant results in social cognition. We analyzed that the small sample size and the short duration of illness in our study, as well as the relatively better functioning of our participants, may explain the lack of significant social cognition impairment in UMFE patients compared to healthy controls. Our study suggests that working memory and attention may be potential candidate biomarkers of schizophrenia. Overall, our findings highlight the importance of assessing cognitive functioning in UMFE patients and developing targeted interventions to improve cognitive outcomes in this population.

### 4.2. PPI deficiency In UMFE and Its correlation with clinical symptoms

This study also found significant deficits in PSSPPI with 60 ms and 120 ms intervals in patients with schizophrenia, with effect sizes significantly larger than those reported in a recent meta-analysis on PPI deficits in chronic schizophrenia (PPI 60 ms (SMD = −0.50) and PPI 120 ms (SMD = −0.44)) (42). Given the abundant evidence showing continuous and selective attention deficits in schizophrenia (43, 44), the PPI experimental paradigm used in this study assessed the subjective attention component, increasing the discriminant validity for the disease with medium to large effect sizes. Scholes et al. (45) reported that attention to auditory stimuli did not decrease the sensitivity of patients to startle stimuli, and the PPI deficits observed in schizophrenia patients resulted from selective attention deficits, which is consistent with our findings. Previous research has shown PPI deficits not only in patients with first-episode schizophrenia (46) but also in those at high clinical risk of psychosis (47) and unaffected first-degree relatives (48).It is noteworthy that modified PPI can be considered a potential biomarker for the disease.

Moreover, there is a correlation between the PSSPPI and PANSS total score, indicating its association with symptom severity of the disease. Dawson et al. (49) also proposed that the impaired attentional modulation of PPI reflects fundamental neurocognitive processes related to thought disorder in schizophrenia. In addition, studies have confirmed that PPI deficits occurring when the prepulse is attended are more strongly associated with symptom severity in the schizophrenia spectrum (50), and that no correlation between symptoms and PPI deficits can be detected in passive attention PPI paradigms in schizophrenia patients (51). Hamm et al. (52) reported that there is a specific relationship between PPI and curative effect. Therefore, modified PPI may serve as an objective predictor of clinical symptom relief. Antipsychotic drug treatment can partially enhance PPI deficits, with atypical drugs, such as quetiapine (53), being more effective. PPI is a useful endophenotype predictor of the disease (54), with close ties to the disease state and potential applications as a predictive indicator for clinical remission. Future research should examine potential influencing factors, such as single nucleotide polymorphisms (SNPs) (55), DNA methylation, childhood trauma, nicotine use, and medication treatment (51), to investigate mechanisms underlying the improvement of PPI defects and better understand the relationship between improved PPI and disease symptoms.

### 4.3. Correlation between the PSSPPI and cognitive function

In this study, a significant correlation was found between PSSPPI and attention/vigilance and social cognition, even after controlling for gender, age, smoking, and years of education. The research team had previously discovered a positive correlation between attention and PSSPPI in patients with chronic schizophrenia, suggesting that attention deficit may be the core problem of PPI deficiency (31). Previous studies using traditional PPI did not find a significant link between PPI at 60 ms and 120 ms and the composite score of MCCB and each domain (56). This may be due to disparities in the inclusion of the disease population and the PPI paradigm. Numerous studies have demonstrated that patients with schizophrenia have deficits in PPI, which may be due to the involvement of several brain regions in the PPI regulation circuit and the pathophysiology of schizophrenia (57). The sensorimotor gating mechanism is controlled by a complex circuit that includes the downlink forebrain pathway (58). Animal studies first associated PPI with the ventral striatum and dopamine (59), which was later presumed to be controlled by the cortico-striato-pallido-pontine circuit (8). This circuit overlaps significantly with the areas linked to the pathological manifestations of schizophrenia, suggesting a behavioral association. Therefore, PPI may be an indicator of higher-order psychopathological injuries.

This study did not find any significant association between PSSPPI and cognitive function in healthy individuals. However, in the UMFE group, a significant correlation was observed between PSSPPI and deficits in attention and working memory, which may be attributed to a core deficit in attention among individuals with schizophrenia. The attention component highlighted by the modified PPI in this study enhanced the effect size of this deficit, indicating a specific relationship between the two. This finding may help explain why a correlation was observed in the patient group but not in the healthy control group.

The study found that PPI deficits were more pronounced at 60 ms intervals than at 120 ms intervals in patients with UMFE. It was also observed that there was a significant association between PPI at 60 ms intervals and higher cognitive functions, such as attention, working memory, and social cognition. However, no such link was found at 120-ms intervals. This suggests that PPI deficiency at 60 ms intervals is more sensitive to the disease and impairment of higher cognitive functions, which aligns with the fact that PPI deficits are most commonly reported at this interval in schizophrenia. (9, 60, 61). Research has discovered that reflection inhibition 60 ms after prepulse appears to be controlled by the process between automatic inhibition and attention-sensitive inhibition (62). In other words, the time domain of this inhibition is at the transition point between the information that is automatically modified and the information that can be influenced at will. Theoretical models have suggested that the transition zone between conscious accessibility and unconscious processing is specifically critical for regulating the content of consciousness, and it may also be a specifically fragile period of psychopathological state (63, 64). This fact implies that gating at 60-ms intervals may be especially critical for the biology of schizophrenia.

## 5. Limitation

This research has several potential limitations. First, the limited sample size of this study may restrict the generalizability of the findings. This may also be one of the reasons why the social cognition domain did not yield any significant results. Second, this study is the first to explore the relationship between modified PPI and cognitive functions in UMFE patients. As these findings are preliminary, none of the results were significant after multiple comparisons. However, this study can serve as a foundation for future research in this area. Another study suggested that there may be differences in the underlying structure of the MSCEIT between individuals with schizophrenia and healthy controls (65). Additionally, it is important to note that the internal consistency of the MSCEIT scale is low in China (66). Moreover, social cognition may mediate information processing speed, attention, and function, suggesting that analyzing the association between a single cognitive dimension and PSSPPI may be insufficient to elucidate the high pathological impairment reflected by PSSPPI (64). Finally, the direct analysis of the relationship between PPI and cognitive function did not consider the potential mediating role of other factors, such as childhood trauma. Future research could use genetic imaging and other technologies to evaluate the specific mechanisms of PPI defects and predict drug efficacy and disease outcomes in prospective cohort studies.

## 6. Conclusion

This study represents the first investigation of the relationship between cognitive functions and modified PPI in Chinese patients with first-episode unmedicated schizophrenia. Modified PPI is considered an index for evaluating attention, cognition, and sensory integration. The study found that the modified PPI includes a subjective attention component, which enhances its discriminant validity for the disease. Moreover, the modified PPI was significantly associated with attention/vigilance, indicating that advanced cognitive function deficits in patients with schizophrenia may be reflected by the modified PPI. Specifically, PSSPPI at 60 ms intervals in UMFE patients was significantly related to clinical symptoms and cognitive function, suggesting that PSSPPI may be a useful tool for evaluating psychopathological symptoms associated with psychosis.

## Data availability statement

The raw data supporting the conclusions of this article will be made available by the authors, without undue reservation.

## Ethics statement

The studies involving human participants were reviewed and approved by Beijing Anding Hospital, Capital Medical University. The patients/participants provided their written informed consent to participate in this study.

## Author contributions

YD: data acquisition, data curation, formal analysis, investigation, methodology, and writing—original draft. QT: data analysis. WH: data acquisition and formal analysis. ZC: data acquisition. ZM: data acquisition. QB: writing—review. FD: funding acquisition, and writing—review and editing. CW: funding acquisition, supervision, and writing—review and editing. All authors contributed to the article and approved the submitted version.

## Funding

This study was funded by the National Natural Science Foundation of China (81971250), Discipline Backbone of High-level Public Health Technical Personnel Training Program of Beijing Municipal Health Commission (2022–3-014), and Beijing Hospitals Authority Youth Programme (QML20191904).

## Conflict of interest

The authors declare that the research was conducted in the absence of any commercial or financial relationships that could be construed as a potential conflict of interest.

## Publisher’s note

All claims expressed in this article are solely those of the authors and do not necessarily represent those of their affiliated organizations, or those of the publisher, the editors and the reviewers. Any product that may be evaluated in this article, or claim that may be made by its manufacturer, is not guaranteed or endorsed by the publisher.
